# Modification of fast-slow differential agar medium for selective isolation of potential starter lactic acid bacteria from cheese

**DOI:** 10.1016/j.mex.2022.101922

**Published:** 2022-11-17

**Authors:** Ziba Güley, Vincenzo Fallico, Tom Beresford

**Affiliations:** a Teagasc Food Research Centre, Moorepark, Fermoy, Co. Cork, Ireland; b Alanya Alaaddin Keykubat University, Department of Food Engineering, Alanya, Antalya, Türkiye

**Keywords:** *Streptococcus*, Selective and Differential Medium, *Lactococcus*, Nalidixic acid

## Abstract

Starter Lactic Acid Bacteria (LAB) are responsible for converting lactose to lactic acid during cheese manufacturing and, as a result, play a critical role in defining the attributes of the final product. There is great interest in isolating novel starter LAB strains to provide alternatives to existing industry cultures or to help enhance the quality and safety of cheeses traditionally made without starter cultures addition [1].

The Fast-Slow Differential Agar (FSDA) medium was developed in 1984 and still remains the standard to rapidly differentiate fast and slow milk-coagulating lactic streptococci and thus avoid screening a large number of isolates for acid production capacity [2]. However, we found that FSDA was unable to selectively isolate fast acid-producing strains from young, traditional, starter-free Izmir Brined Tulum cheeses, due to the presence of a diverse microbiome including Non-Starter LAB and spoilage Gram-negative microbiota [1, 3].

Here, we describe a modified FSDA (mFSDA) with increased selectivity and recovery efficiency towards lactic streptococci, which was successfully used to rapidly isolate potential starters from Tulum cheeses [1] and could similarly outperform FSDA in raw milk cheeses and other varieties containing high levels of “background” microbiota. The main differences between FSDA and mFSDA media consist in the presence of nalidixic acid, ascorbic acid and yeast extract in mFSDA. These targeted additions provide mFSDA with a two-prong selectivity that (I) suppresses unwanted microbiota, and (II) increases the recovery efficiency of lactic streptocci adept to using milk nutrients. Specifically:

• Nalidixic acid is an antibiotic that primarily inhibit Gram-negative bacteria [4].
• Ascorbic acid and yeast extract stimulate the growth of lactic streptococci [5] and were added to complement skim milk in creating an environment favoring the growth of lactose-positive, casein peptides-utilizing LAB.
• The pH indicator bromocresol purple enabled the chromogenic discrimination between LAB with different acid production capability.

## Graphical abstract

**Figure:**
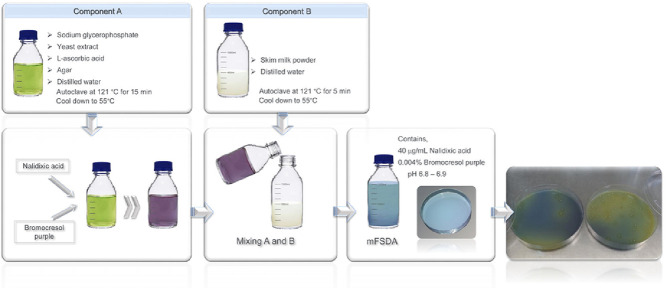

Specifications table

**Table:** 

Subject area:	Food Science
More specific subject area:	Food Microbiology
Name of your method:	Modified Fast-Slow Differential Agar (mFSDA) Medium
Name and reference of original method:	Huggins, A. R. & Sandine, W. E. (1984). “Differentiation of Fast and Slow Milk-Coagulating Isolates in Strains of Lactic Streptococci”, Journal of Dairy Science, 67(8), 1674-1679. https://doi.org/10.3168/jds.S0022-0302(84)81491-5
Resource availability:	None

## Method details

**Fig. 1:**
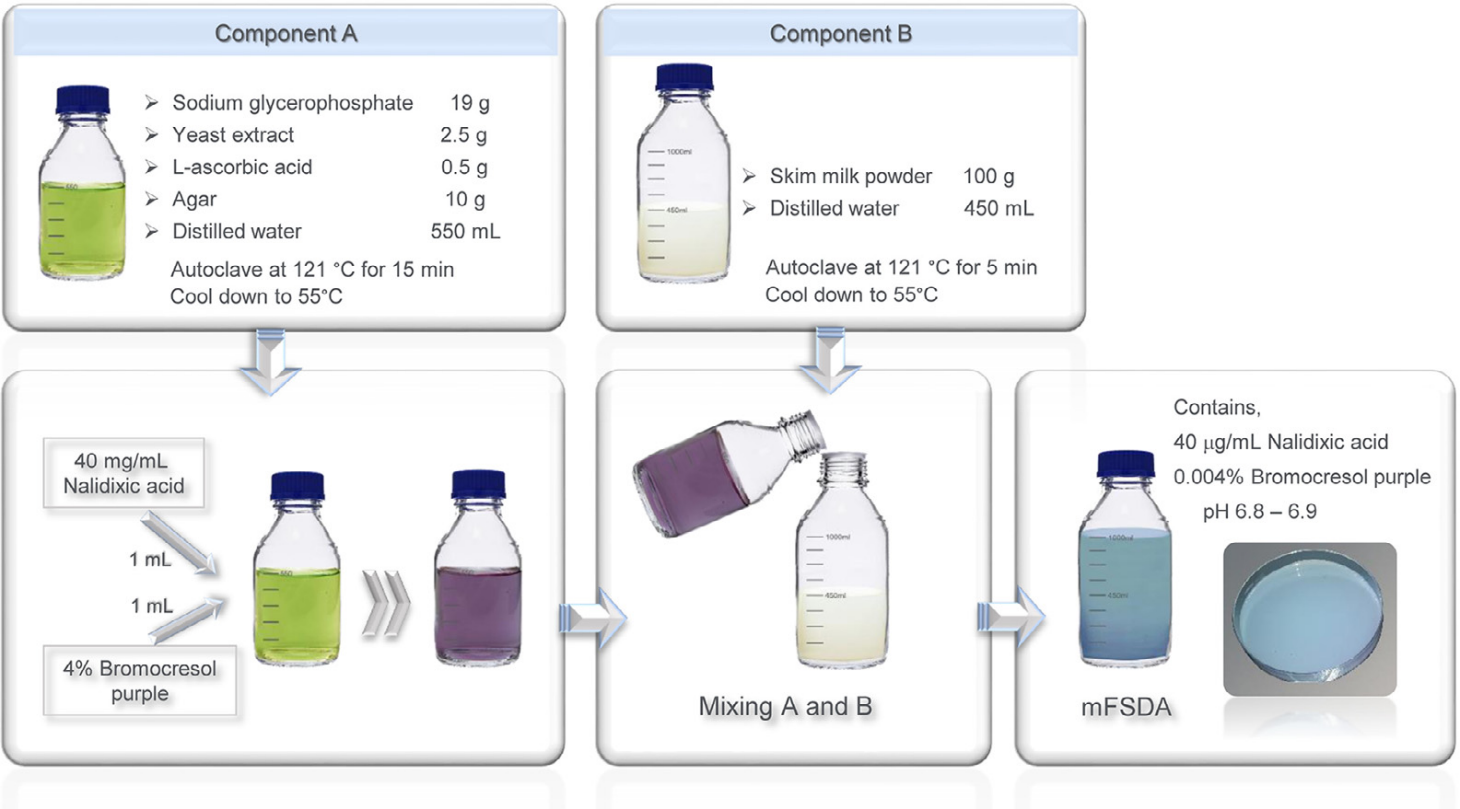
Preparation steps of modified FSDA (mFSDA) medium.

The composition of mFSDA medium is provided in Table 1. The medium was prepared as two components, A and B, according to the workflow depicted in Fig. 1. Component A was made by dissolving 19 g of sodium glycerophosphate (catalog no. G5422, Sigma-Aldrich), 2.5 g of yeast extract (catalog no. LP0021, Oxoid), 0.5 g of L-ascorbic acid (catalog no. A7506, Sigma-Aldrich), and 10 g of agar in 550 mL of distilled water. Component B was made by dissolving 100 g of skim milk powder (catalog no. LP0031, Oxoid) in 450 mL of distilled water. Components A and B were sterilized separately by autoclaving at 121°C for 15 min and at 121°C for 5 min, respectively. Sterilized components were cooled to 55°C in a water bath, mixed together by gentle swirling, followed by addition of 40 μg/mL nalidixic acid sodium salt (NA, catalog no. N4382, Sigma-Aldrich) and 0.004% (w/v) bromocresol purple (catalog no. 1.03025, Merck) and poured into petri dishes (Fig. 1). It is important to use nalidixic acid sodium salt to prepare the nalidixic acid solution, as the salt-free antibiotic is insoluble in distilled water. Solution was filter-sterilized before use.

**Table 1:** Composition of original FSDA and modified FSDA.

Component	Composition (g/L or mL/L*)		Function [2,^4^,5]
	Original FSDA [2]	Modified FSDA	
Sodium glycerophosphate	19	19	Buffering agent
Yeast extract	-	2.5	Growth stimulator
L-ascorbic acid	-	0.5	Growth stimulator
Nalidixic acid	-	0.04	Inhibition of spoilage G(+) and G(-) bacteria
Skim Milk Powder	100	100	Nutrient source
Litmus	1	-	Chromogenic discrimination
Bromocresol purple 4% Solution	-	1 mL*	Chromogenic discrimination
Agar	10	10	Solidifying agent

For mFSDA bromocresol purple was chosen over litmus as it was reported to be equivalent to litmus by the developers of the FSDA medium [2].

## Method validation

The mFSDA medium was initially validated using a bank of 27 Gram-positive and Gram-negative bacteria isolated from raw milks, dairy plants and cheeses during previous projects and stored in the Teagasc DPC culture collection (Table 2). Results demonstrated the ability of mFSDA to support the growth of all LAB investigated, except for *Schleiferilactobacillus harbinensis*, while most potential spoilage microorganisms were unable to grow on mFSDA except for *Bacillus subtilis*. Most of the LAB species tested also produced acid, except for some species normally associated with the Non-Starter LAB microbiota, such as *Levilactobacillus brevis, Lentilactobacillus parabuchneri* and *Latilacobacillus curvatus*.

**Table 2:** Growth and acidification ability on mFSDA medium of several LAB*, potential spoilage Gram-positive^‡^ and Gram-negative^†^ bacterial species, of dairy and raw milk origin.

Species	Strain ID	Growth	Acidification
*Lactococcus lactis subsp*. lactis*	303	+	+
*Lactococcus lactis subsp*. lactis*	227	+	+
*Streptococcus thermophilus	DPC 421	+	+
*Streptococcus thermophilus	1796	+	+
*Streptococcus pasteurianus	C28T2	+	+
*Enterococcus faecalis	C29T1	+	±
*Enterococcus durans	SMS22-T1	+	+
*Leuconostoc mesenteroides	BAT 10	+**^w^**	+
*Lactobacillus helveticus	R1-B2	+	+
*Lactobacillus delbrueckii	4R200	+	+
*Lentilactobacillus parabuchneri	03H-08	+**^w^**	–
*Levilactobacillus brevis	SIL1T2	+**^w^**	–
*Lactiplantibacillus plantarum	SIL1T4	+	+
*Lacticaseibacillus paracasei	3H18	+	+
*Loigolactobacillus coryniformis	56B1	+**^w^**	–
*Latilactobacillus curvatus	70A	+**^w^**	–
*Schleiferilactobacillus harbinensis	4R19	–	–
*Lacticaseibacillus zeae	3R3	+	+
*Companilactobacillus nodensis	1H8	+**^w^**	–
*^‡^Bacillus subtilis*	S9BH2	+	+
*^‡^Kurthia gibsonii*	SW20-H1	–	–
*^†^Pseudomonas fragi*	BA9	–	–
*^†^Raoultella ornithinolytica*	SIL1H1	–	–
*^†^Raoultella terrigena*	S16	–	–
*^†^Citrobacter freundii*	BT15F	–	–
*^†^Acinetobacter baumannii*	SW21-H2	–	–
*^†^Obesumbacterium proteus*	BAH16	–	–

+: Good growth on mFSDA/ Good acid production on mFSDA, +^w^**:** weak growth, ±: weak acid production

–: No growth on mFSDA/ No acid production on mFSDA

Following this successful validation, we tested the ability of mFSDA to isolate potential starter streptococci strains from young Izmir Brined Tulum cheeses. Due to the absence of starter addition during manufacturing, young samples of this traditional cheese variety can accumulate spoilage Gram-negative microbiota [1,3] and therefore presented an ideal challenge for mFSDA.

**Fig. 2:**
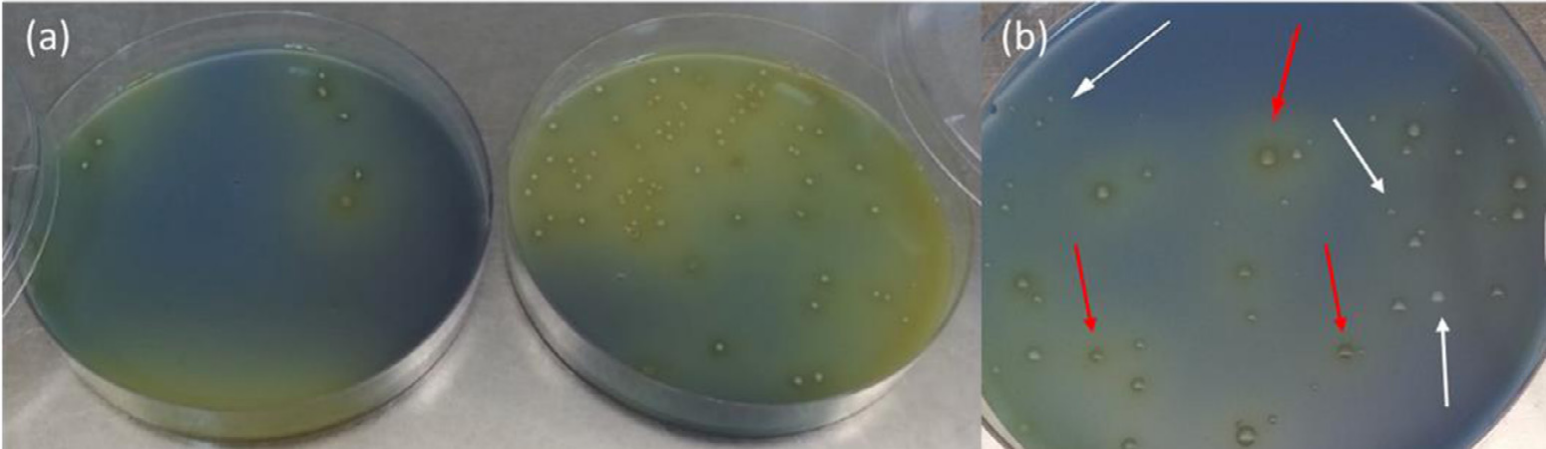
mFSDA plates showing a mix of fast and slow acid-producing bacteria from Izmir Brined Tulum cheese (2a, 2b). Zoomed section of mFSDA plate (2b) showing the different colony morphologies of fast acidifiers (red arrows) and slow acidifiers (white arrows).

A key trait of a starter culture for cheese production is the ability to grow rapidly in milk and reduce the pH to ≤ 5.3 in 6 hours. On the other hand, non-starter LAB grow slowly in milk and do not cause such a pH reduction as they are generally lactose-negative and use peptides and free amino acids released from casein by the action of the starter LAB and residual chymosin in the cheese as their nitrogen source [6]. mFSDA incorporates an acid/base indicator (bromocresol purple) that changes color from blue to yellow when the agar pH is 5.2 or lower. Consequently, potential starter streptococci, fast-acidifying bacteria would appear as yellow colonies on mFSDA, while non-starter slow acidifiers should form white colonies. Our tests confirmed this, but also revealed that when high numbers of fast-acidifying colonies are present on a plate, the acid produced tends to defuse throughout the plate turning the medium yellow (Fig. 2a). This can make it difficult to establish with confidence the colony responsible for the acid production and can lead to selection of false positives. To avoid this, fast acidifiers should only be selected from plates with less than 50 colonies (Fig. 2b). In such a case, fast-acidifying bacteria appear as large, yellow colonies surrounded by a clearing halo indicative of their capacity to hydrolyze milk caseins (Fig. 2b, red arrows). In contrast, slow acidifiers mostly appear as smaller, white colonies with no clearing halo around them (Fig. 2b, white arrows). This facilitates the identification of fast-acidifying isolates that could have potential as starter cultures.

In agreement with this, results of the related study [1] found that the majority of the Izmir Brined Tulum cheese isolates selected using mFSDA, including strains of *Streptococcus infantarius* subsp. *infantarius* and *Streptococcus macedonicus*, were confirmed to be good acid producers when grown in 10% reconstituted skim milk.

In conclusion, the newly developed mFSDA medium affords the same rapid and direct selection of fast acid-producing strains of lactic streptococci as the original FSDA medium while enhancing the recovery efficiency in environments containing background Gram-negative microbiota.

## Declaration of Competing Interest

The authors declare that they have no known competing financial interests or personal relationships that could have appeared to influence the work reported in this paper.

## Data Availability

No data was used for the research described in the article.
